# Radiological and Histological Findings of Primary Pleomorphic Rhabdomyosarcoma Arising from the Mandibular Gingiva: A Case Report and Literature Review

**DOI:** 10.3390/diagnostics16162631

**Published:** 2026-08-19

**Authors:** Yun Hwa Shim, Hye Jin Baek, Jieun Roh, Seung Kug Baik, Kwang Ho Choi, Tae Un Kim, Hwaseong Ryu

**Affiliations:** 1 Department of Radiology, Research Institute for Convergence of Biomedical Science and Technology, Pusan National University Yangsan Hospital, Pusan National University School of Medicine, 20 Geumo-ro, Mulgeum-eup, Yangsan-si 50612, Gyeongsangnam-do, Republic of Korea; 2 Department of Thoracic and Cardiovascular Surgery, Research Institute for Convergence of Biomedical Science and Technology, Pusan National University Yangsan Hospital, Pusan National University School of Medicine, 20 Geumo-ro, Mulgeum-eup, Yangsan-si 50612, Gyeongsangnam-do, Republic of Korea

**Keywords:** rhabdomyosarcoma, pleomorphic rhabdomyosarcoma, gingiva, oral cavity, mandible, computed tomography, magnetic resonance imaging

## Abstract

**Background**: Pleomorphic rhabdomyosarcoma (RMS) is a rare, adult-predominant high-grade sarcoma that usually arises in the deep soft tissues of the extremities. Primary oral pleomorphic RMS is exceptionally rare, and detailed CT and MRI characteristics of oral pleomorphic RMS remain sparsely documented. **Case Presentation**: A 66-year-old woman presented with a two-month history of lower anterior tooth pain and progressive mandibular swelling, initially misdiagnosed and treated as a dental infection. CT and MRI revealed a 3.8-cm heterogeneously enhancing mass centered in the mandibular gingiva, with aggressive cortical destruction, diffusion restriction, and anterior floor-of-mouth extension; oral cavity cancer (squamous cell carcinoma) was initially favored on imaging. The patient underwent wide excision with segmental mandibulectomy and fibular osteocutaneous free-flap reconstruction. Histopathologic examination confirmed a high-grade pleomorphic RMS with immunoreactivity for desmin and MyoD1. The patient received adjuvant chemotherapy and radiotherapy, with no recurrence at 8-month follow-up. **Conclusions**: Pleomorphic RMS of mandibular gingiva may be mistaken clinically for odontogenic infection and radiologically for squamous cell carcinoma. Although imaging findings are nonspecific, CT and MRI are essential for defining mandibular and floor-of-mouth involvement and planning resection; definitive diagnosis requires histopathologic and immunohistochemical confirmation.

## 1. Introduction

Rhabdomyosarcoma (RMS) is a malignant mesenchymal neoplasm exhibiting skeletal muscle differentiation, subclassified into four distinct types: embryonal, alveolar, spindle cell/sclerosing, and pleomorphic [1,2]. Among its variants, pleomorphic RMS is an uncommon, adult-predominant, high-grade sarcoma that most frequently arises in the deep soft tissues of the extremities; oral cavity involvement is exceptional, accounting for only a minority of cases even in large clinicopathologic series [3]. Although RMS occasionally occurs in the head and neck region, adult head and neck RMS remains uncommon overall [4,5,6].

To date, primary oral or perioral pleomorphic RMS is particularly rare, with only sporadic cases documented at sites such as the gingiva, upper lip, maxillary vestibule, and tongue [3,7,8,9,10,11,12]. Because the majority of these prior reports focused predominantly on clinical presentations or histopathologic outcomes, detailed CT and MRI descriptions of oral pleomorphic RMS remain scarce. Documenting these radiological characteristics is clinically relevant, as the lesion can frequently masquerade as more common inflammatory or carcinomatous processes. Herein, we report a rare case of primary pleomorphic RMS arising from the mandibular gingiva in a 66-year-old woman, presenting as a locally aggressive, bone-destructive oral mass, and comprehensively describe its CT, MRI, and histopathologic findings with a focused review of the literature.

## 2. Case Report

A 66-year-old female presented with a two-month history of pain in the lower anterior teeth and progressive swelling of the anterior mandibular region. At an outside dental clinic, the symptoms had been attributed to an odontogenic infection; she underwent extraction of the involved tooth followed by antibiotic therapy. However, the swelling continued to progress despite treatment, prompting referral to our tertiary hospital for further evaluation.

**Figure 1:**
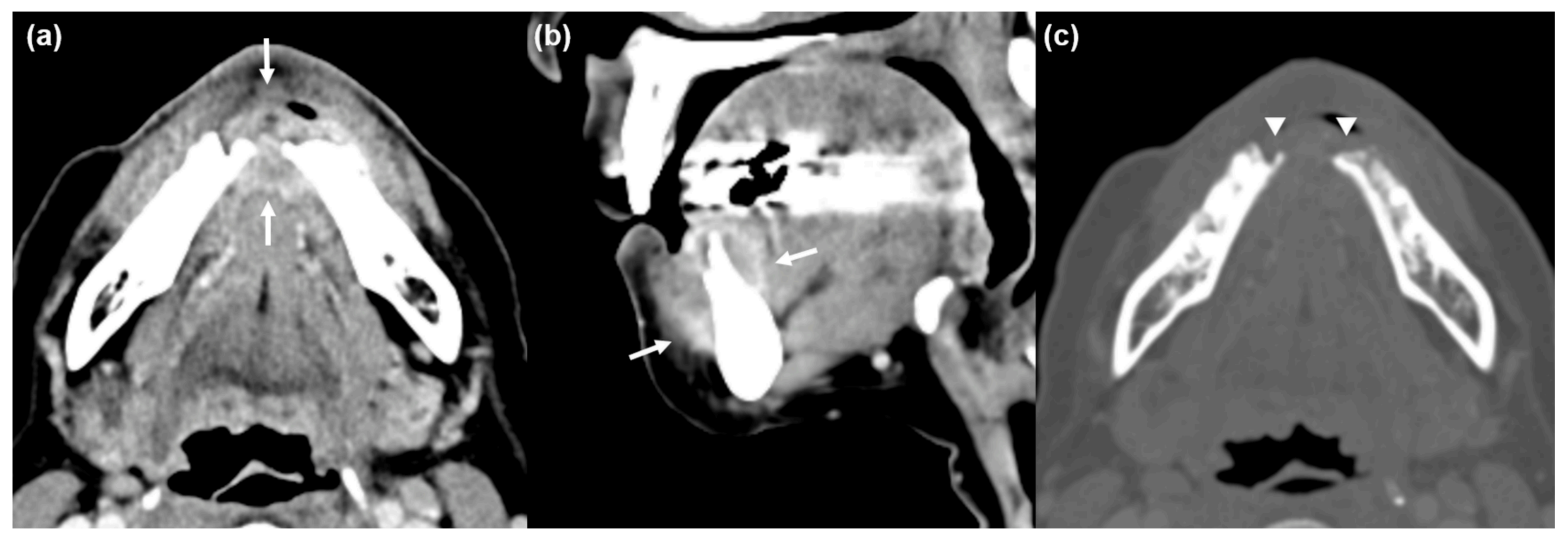
CT findings. (**a**) Axial contrast-enhanced CT image shows a heterogeneously enhancing soft-tissue gingival mass at the mandibular symphysis with focal extension to the anterior floor of the mouth (arrows). (**b**) Sagittal contrast-enhanced CT image shows bidirectional tumor extension across the mandible (arrows). (**c**) Axial bone-window CT images demonstrate cortical bony destruction of the mandible (arrowheads).

**Figure 2:**
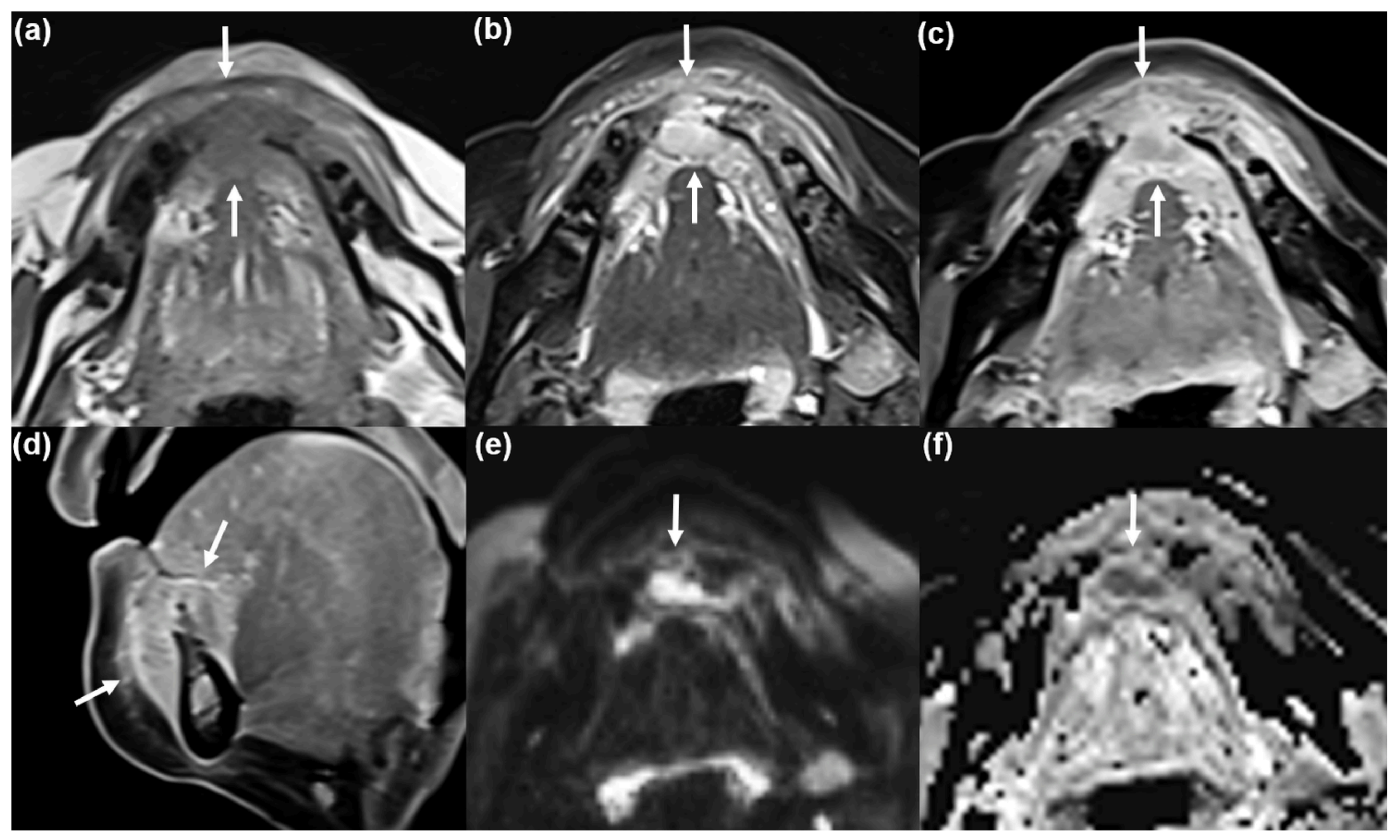
MRI findings. (**a**) Axial T1-weighted image shows a hypointense soft-tissue gingival mass with bone involvement at the mandibular symphysis. (**b**) Axial fat-suppressed T2-weighted image shows inhomogeneous high signal intensity of the lesion with focal tumor extension to the anterior floor of the mouth (in particular, sublingual glands). (**c**,**d**) Axial (**c**) and sagittal (**d**) fat-suppressed contrast-enhanced T1-weighted images show heterogeneous enhancement of the tumor with abutment of the adjacent floor of mouth musculature. (**e**,**f**) Axial diffusion-weighted image (DWI) (**e**) and the corresponding apparent diffusion coefficient (ADC) map (**f**) demonstrate diffusion restriction within the lesion, with a mean ADC value of 0.87 ± 0.23 × 10^−3^ mm^2^/s. Arrows in the images indicate the lesion.

Initial panoramic radiography and cone-beam CT revealed an ill-defined radiolucent osteolytic lesion involving the mandibular symphysis and adjacent alveolar process. Contrast-enhanced neck CT demonstrated a heterogeneously enhancing soft-tissue mass measuring approximately 3.8 cm in maximum diameter, centered at the gingiva overlying the mandibular symphysis, with aggressive cortical destruction of the underlying mandible and extension into the anterosuperior floor of the mouth (FOM) (Figure 1). No definite cervical lymphadenopathy was identified. On subsequent contrast-enhanced neck MRI, the tumor showed inhomogeneous T2 hyperintensity, diffusion restriction (mean ADC, 0.87 ± 0.23 × 10^−3^ mm^2^/s), and heterogeneous enhancement, with disruption of the mandibular cortex and abutment of the adjacent FOM musculature and sublingual gland (Figure 2). Based on the clinical history and imaging findings, oral cavity squamous cell carcinoma (SCC) arising from the mandibular gingiva was considered the leading initial radiologic diagnosis. Systemic staging with chest and abdominal CT showed no evidence of another primary malignancy or distant metastasis. Whole-body 18F-FDG PET/CT demonstrated no abnormal FDG-avid lesion outside the oral cavity tumor, supporting a primary gingival origin. Prior to referral to our institution, an incisional biopsy had been performed at an outside university hospital and demonstrated a malignancy with spindle-cell morphology. After referral to our institution, a second incisional biopsy was performed before definitive surgery and was interpreted as a sarcoma of uncertain type, showing only a few atypical spindle cells in a background of extensive necrosis. Thus, although the two preoperative biopsies established the presence of a malignant spindle-cell/sarcomatous neoplasm, definitive histologic subtyping was not possible.

**Figure 3:**
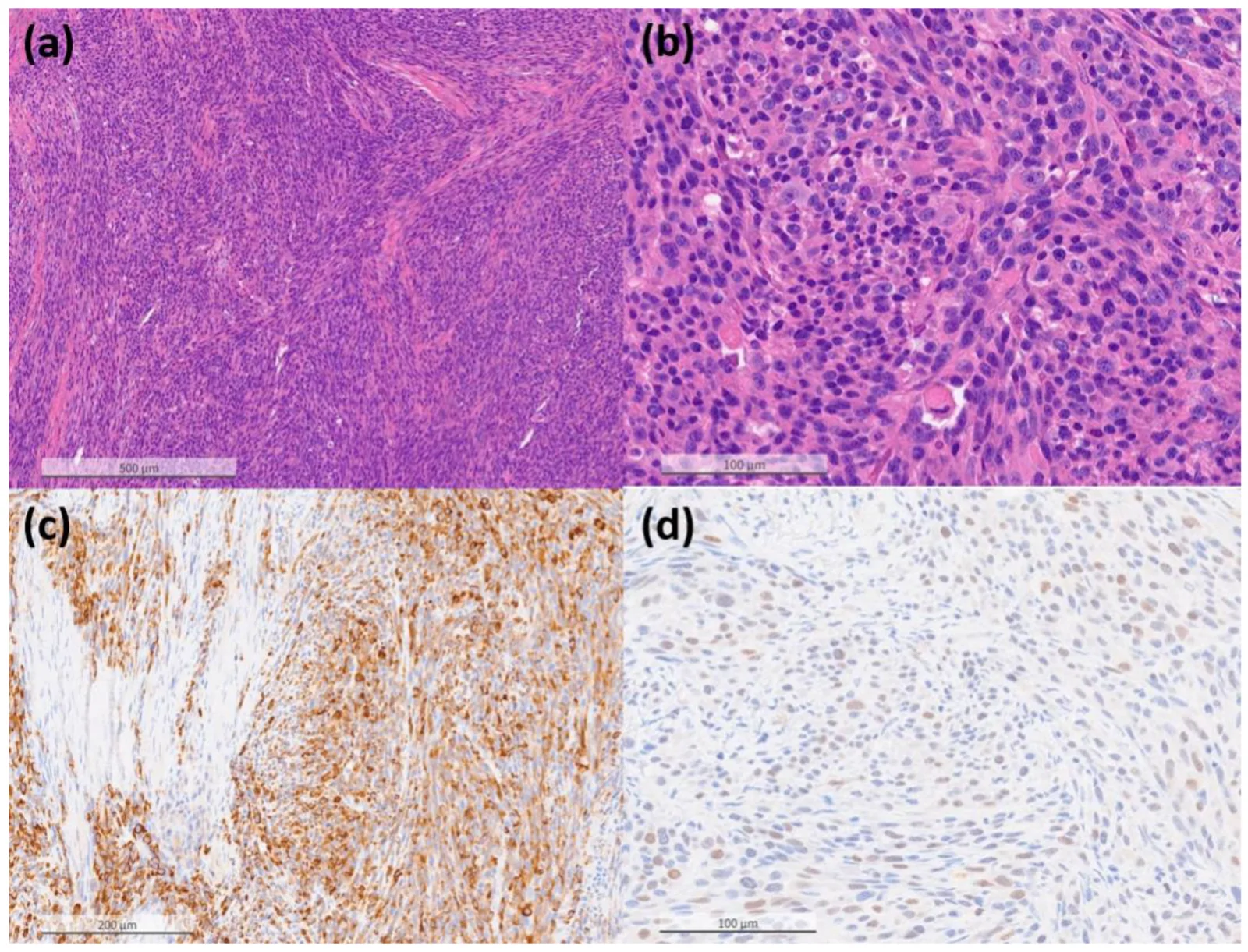
Histopathologic findings of the excised tumor. (**a**,**b**) Hematoxylin and eosin (H&E)-stained sections. (**a**) Low-power view (×50) reveals tumor cells arranged in sheets and fascicles. (**b**) High-power view (×200) shows large atypical polygonal, spindle-shaped, and rhabdoid eosinophilic tumor cells, including occasional multinucleated cells. (**c**,**d**) Immunohistochemical staining demonstrates positivity for desmin (×100) (**c**) and nuclear positivity for MyoD1 (×200) (**d**) in the tumor cells.

The patient underwent wide excision with segmental mandibulectomy and fibular osteocutaneous free-flap reconstruction. Histopathological examination showed a high-grade pleomorphic sarcoma with tumor cells arranged in sheets and fascicles on low-power hematoxylin and eosin (H&E) staining (×50) (Figure 3a). At higher magnification, the tumor was composed of large atypical polygonal, spindle-shaped, and rhabdoid eosinophilic cells, with occasional multinucleated forms (H&E, ×200) (Figure 3b). Immunohistochemistry demonstrated positivity for desmin and nuclear positivity for MyoD1 in the tumor cells (Figure 3c,d) whereas myogenin was negative. Pancytokeratin (AE1/AE3 cocktail) showed focal positivity. Extensive examination of the resection specimen revealed no conventional SCC component, surface epithelial dysplasia, or carcinoma in situ. In conjunction with the pleomorphic histomorphology and myogenic immunophenotype, these findings supported the diagnosis of high-grade pleomorphic RMS. Ancillary molecular testing, including FOXO1 rearrangement analysis, was not performed as part of the clinical diagnostic workup. The patient subsequently received six cycles of adjuvant doxorubicin–ifosfamide chemotherapy and radiotherapy to a total dose of 66 Gy in 33 fractions. At the latest follow-up, 8 months after surgery, no evidence of recurrence was detected.

## 3. Discussion

The present case highlights the diagnostic difficulty of pleomorphic RMS arising in an unusual oral site, adding to the sparse clinicoradiologic literature on this entity by documenting aggressive mandibular gingival involvement with cortical bone destruction and FOM extension. To place this presentation in perspective, we summarize previously reported primary oral and perioral pleomorphic RMS cases with extractable clinicoradiologic information in chronological order of publication in Table 1 [3,7,8,9,10,11,12]. Because this report was not intended as a formal systematic review, Table 1 was constructed using targeted searches of PubMed and Google Scholar, supplemented by a manual search of the reference lists of relevant articles. The searches combined the term “pleomorphic rhabdomyosarcoma” with “oral,” “gingiva” or “gingival,” “tongue,” “lip,” “maxilla,” and “mandible.” Reports were included if they described primary oral or perioral pleomorphic RMS with extractable case-level clinical information; imaging, histopathologic/immunohistochemical, treatment, and outcome data were recorded when available. Duplicate or overlapping cases, non-pleomorphic RMS, non-oral/perioral primary tumors, metastatic lesions to the oral cavity, and reports lacking sufficient case-level information were excluded.

Primary oral soft-tissue and jawbone sarcomas are rare as a broader disease group, further underscoring the unusual nature of pleomorphic RMS arising from the mandibular gingiva [13]. In the present case, the initial clinical impression was an odontogenic inflammatory process. Given the extreme rarity of oral pleomorphic RMS and the much greater frequency of odontogenic inflammatory disease, this was a reasonable initial consideration rather than a diagnostic pitfall. The patient presented with lower anterior tooth pain and mandibular swelling, underwent tooth extraction and antibiotic therapy at a local clinic, and was referred only after the swelling progressed. This clinical course emphasizes the importance of reconsidering the initial diagnosis when gingival swelling persists or progresses despite appropriate dental treatment. This pattern is highly instructive; pleomorphic RMS in the oral cavity can closely mimic common dental or reactive conditions, including inflammatory, proliferative, or endodontic–periodontal lesions [14,15,16]. Therefore, persistent or progressive gingival swelling in an adult should not be attributed to dental infection alone, particularly when symptoms fail to resolve after extraction or antibiotic therapy.

Radiologically, oral cavity SCC was the leading initial consideration. In an older adult, a destructive oral cavity mass centered in the gingival or FOM region would reasonably raise concern for SCC because of its predominance among oral malignancies [17]. In this scenario, multimodal imaging was critical: CT demonstrated aggressive mandibular cortical destruction and defined the extent of bone involvement, while MRI characteristically delineated soft-tissue spread into the FOM and tumor proximity to adjacent musculature. Thus, imaging appropriately indicated an aggressive oral malignancy but was not sufficiently specific to distinguish SCC from a rare mesenchymal malignancy such as pleomorphic RMS.

The differential diagnosis of a mandibular-invading oral cavity mass should therefore remain broader than SCC alone. Radiologists and clinicians should also consider malignant odontogenic tumors, extranodal lymphoma, and primary oral soft-tissue or jawbone sarcomas, guided by the lesion epicenter, relationship to dental structures, mucosal involvement, enhancement pattern, nodal status, and clinical course [13,18,19]. On imaging, pleomorphic RMS may be indistinguishable from other aggressive soft-tissue malignancies presenting as bone-destructive oral cavity masses. Nevertheless, marked T2 hyperintensity and diffusion restriction in an aggressively bone-destructive soft-tissue mass may serve as imaging clues that prompt consideration of RMS or another mesenchymal malignancy rather than SCC alone [20]. These findings should be regarded as suggestive rather than specific, given the substantial imaging overlap with oral SCC.

Although the imaging literature dedicated to oral or gingival pleomorphic RMS is lacking, available reports on adult head and neck or sinonasal RMS suggest that these tumors typically appear as an aggressive soft-tissue mass with nonspecific CT and MRI characteristics, including variable enhancement, diffusion restriction, and local invasion, which may overlap with other head and neck malignancies [21,22]. Imaging alone is therefore unlikely to establish a definitive preoperative diagnosis of pleomorphic RMS. Nevertheless, CT and MRI remain essential for defining disease extent—including cortical bone destruction, FOM involvement, relationship to adjacent soft-tissue structures, and cervical nodal status—and for guiding surgical planning.

Consequently, histopathologic and immunohistochemical correlation is indispensable for the definitive diagnosis of pleomorphic RMS. In this case, the resected tumor showed a high-grade pleomorphic sarcoma composed of markedly atypical spindle to polygonal cells with abundant eosinophilic cytoplasm, frequent mitoses, and bizarre tumor giant cells. Immunoreactivity for desmin and nuclear expression of MyoD1 supported skeletal muscle differentiation, whereas myogenin was negative. Myogenin expression may be variable in pleomorphic RMS and its absence does not preclude the diagnosis [1,2,3]. Accordingly, the diagnosis of pleomorphic RMS requires integration of characteristic pleomorphic morphology with evidence of skeletal muscle differentiation and exclusion of relevant mimics. Although focal pancytokeratin expression may raise consideration of sarcomatoid carcinoma, aberrant keratin expression has been documented in adult-type RMS; therefore, focal keratin positivity should be interpreted in conjunction with tumor morphology and the overall myogenic immunophenotypic profile [23]. Nevertheless, because dedicated p40, p63, and CK5/6 immunostaining was not performed, sarcomatoid carcinoma cannot be conclusively excluded on immunohistochemical grounds alone. In the present case, the diagnosis of pleomorphic RMS was therefore based on the integrated histomorphologic and myogenic immunophenotypic findings, together with the absence of a conventional SCC component, surface epithelial dysplasia, or carcinoma in situ in the extensively examined resection specimen. This distinction is clinically important; while oral SCC, odontogenic malignancies, lymphoma, and other pleomorphic sarcomas share overlapping clinicoradiologic appearances, their therapeutic strategies diverge significantly.

The occurrence of pleomorphic RMS in the gingiva, a site without intrinsic skeletal muscle, raises a question regarding its cell of origin. Gingival mesenchymal stem/progenitor cells include cranial neural crest- and mesoderm-derived populations [24,25], and experimental studies have demonstrated that gingiva-derived mesenchymal stem cells can undergo myogenic differentiation under specific in vitro conditions [26]. However, these experimental observations are indirect and do not establish the cell of origin of gingival pleomorphic RMS. Recent genomic studies characterize pleomorphic RMS as a genomically complex sarcoma molecularly distinct from embryonal RMS, with recurrent disruption of TP53/RB1-related cell-cycle pathways [27,28]. However, no recurrent molecular alteration currently defines pleomorphic RMS, and ancillary molecular testing has limited utility for its subclassification [28]. Molecular testing was not performed in the present case. Thus, malignant transformation of a resident multipotent mesenchymal progenitor followed by aberrant myogenic lineage activation may represent a biologically plausible hypothesis; however, this model remains unproven and should not be interpreted as an established mechanism of tumorigenesis [24,25,26,27,28].

The optimal treatment for pleomorphic RMS has not been standardized due to its rarity. Adult head and neck RMS is generally managed using a multimodal strategy that includes wide surgical excision, radiotherapy, and systemic chemotherapy, tailored to tumor extent, resectability, margin status, histologic subtype, and patient factors [29]. Among systemic regimens, vincristine, actinomycin D, and cyclophosphamide (VAC) has been commonly used in RMS and has also been reported in adult oral RMS cases [12]. For adult head and neck RMS, definitive radiotherapy doses of 66–70 Gy in 2-Gy fractions have been reported for embryonal/alveolar subtypes, whereas pleomorphic tumors have generally been managed with surgery with or without radiotherapy [29]. In the present case, multimodal treatment achieved short-term disease control, with no recurrence detected at 8 months. However, this follow-up period remains relatively short, and continued long-term surveillance is required to assess the durability of local and systemic control.

## 4. Conclusions

We present an exceedingly rare case of primary pleomorphic RMS arising from the mandibular gingiva in an elderly patient, which closely mimicked both a treatment-resistant odontogenic infection and oral cavity SCC on clinical and radiologic grounds. When radiologists and clinicians encounter an aggressive oral cavity mass with mandibular cortical destruction—particularly in adult patients with a refractory clinical course despite antibiotic treatment—rare mesenchymal malignancies, including pleomorphic RMS, should be considered in the differential diagnosis alongside SCC. Although CT and MRI are essential for defining local extent and guiding surgical planning, definitive diagnosis requires histopathologic and immunohistochemical confirmation. Enhanced clinical awareness of this rare entity is crucial to avoid diagnostic delays and facilitate timely, appropriate multimodal management.

## Figures and Tables

**Table 1:** Reported primary oral and perioral pleomorphic rhabdomyosarcoma cases with extractable clinicoradiologic information.

Reported Case	Age/ Sex	Site	Size/Clinical Presentation	Imaging Modality and Reported Imaging Findings	Bone Involvement	Histopathologic/IHC Findings	Treatment/Outcome
**Kaloyannides, 1969** [7]	51/M	Right mandibular gingiva	3.5 × 1.8 × 1.4 cm/mandibular swelling, impaired mastication, pain and hemorrhage on pressure	X-ray: no osseous lesion	No	Historical pre-modern IHC era	Wide excision and radiotherapy for local recurrence; no recurrence reported at unspecified follow-up
**Piattelli, 1991** [8] ^†^	Adult	Upper lip	NA	NA	NA	Pleomorphic RMS of the upper lip; perioral rather than intraoral site	NA
**Furlong et al., 2001** [3]	77/M	Floor-of-mouth	5 cm/Rapidly growing tumor with painful swelling	NA	NA	Desmin, myoglobin, MyoD1, myf3, myf4 and MSA positive; SMA negative	Died of disease at 6 months
**Singh et al., 2013** [9]	10/F	Left maxillary vestibule/upper jaw, extending from left palate to buccal vestibule	3 × 5 cm/facial swelling, pain, mastication and swallowing difficulty	CT: ill-defined destructive lesion involving the maxilla, maxillary sinus, floor of left orbit, with extension to adjacent structures	Yes	Desmin and Ki-67 positive; CK, S100 and SMA negative	Surgery and chemotherapy; died after 3 cycles
**Amtha et al., 2019** [10]	42/M	Posterior right lateral border of tongue	2.5 × 0.3 cm/nonhealing painful ulcer for 4 months; palpable right submandibular lymph node; weight loss	No detailed CT/MRI findings reported; radiologic workup for occult primary/metastasis reportedly negative	NA	SMA and AE1/AE3 strongly positive, desmin and S100 occasionally positive; Ki-67 index 80%; myogenin negative	Wide excision with regional neck dissection, chemotherapy and radiotherapy; no new tumor at 6 months
**Chatterjee et al., 2024** [11]	30/F	Maxillary gingiva, maxillary alveolar process, involving alveolar and buccal mucosa	Size: NA/soft gingival mass with pain and swelling	OPG: extensive bone loss in maxillary anterior region with faint soft tissue shadow. CT/CBCT: ill-defined heterogeneously enhancing soft tissue mass with extensive destruction of maxillary dentoalveolar and palatal bone	Yes	Desmin, SMA, myogenin and MyoD1 positive; S100 negative	Chemotherapy, wide maxillary excision and radiotherapy; alive at 8 months follow-up
**Sideris et al., 2025** [12]	49/M	Base of tongue	4.9 × 4.3 × 4.2 cm/halitosis, dysphagia, foreign body sensation, “hot potato voice”, and airway narrowing	MRI: large tongue-base mass displacing the uvula and extending to epiglottis; recurrence described as 1.6 × 2 cm homogeneously enhancing lesion with diffusion restriction	No	Desmin diffusely positive, myogenin strong focal positive, MyoD1 weak focal positive	Surgical resection; early recurrence; second surgery and radiotherapy; no recurrence at 12 months
**Present case**	66/F	Mandibular gingiva, median mandibular body/mandibular symphysis, with anterior floor-of-mouth extension	3.8 cm heterogeneously enhancing mass; initially treated as dental infection; progressive swelling despite extraction and antibiotics	CT: heterogeneously enhancing gingival mass with aggressive mandibular cortical destruction and floor-of-mouth extension. MRI: T2 heterogeneous hyperintensity, diffusion restriction, heterogeneous enhancement, cortical disruption, floor-of-mouth musculature abutment	Yes	Desmin and MyoD1 positive	Wide excision with segmental mandibulectomy and flap reconstruction; adjuvant chemotherapy and radiotherapy; no recurrence at 8 months

Note. Cases were identified through targeted PubMed and Google Scholar searches supplemented by manual searching of reference lists, as described in the Discussion. The cases are arranged in chronological order of publication; accordingly, reference numbers appear out of sequential order. NA, not applicable. ^†^ Data for Piattelli (1991) [8] were extracted from the published abstract, as the full-text article was unavailable for review.

## Data Availability

The original contributions presented in this study are included in the article. Further inquiries can be directed to the corresponding author.
